# Complete Genome Sequence of a *Staphylococcus epidermidis* Bacteriophage Isolated from the Anterior Nares of Humans

**DOI:** 10.1128/genomeA.00549-14

**Published:** 2014-08-07

**Authors:** Vijay H. Aswani, Denise M. Tremblay, Sylvain Moineau, Sanjay K. Shukla

**Affiliations:** a Department of Internal Medicine & Pediatrics, Marshfield Clinic, Marshfield, Wisconsin, USA; b Groupe de Recherche en Ecologie Buccale and Félix d’Hérelle Reference Center for Bacterial Viruses, Faculté de Médecine Dentaire, Université Laval, Quebec City, Quebec, Canada; c Département de Biochimie, de Microbiologie et de Bio-informatique, Faculté des Sciences et de Génie, Université Laval, Quebec City, Quebec, Canada; d Center for Human Genetics, Marshfield Clinic Research Foundation, Marshfield, Wisconsin, USA

## Abstract

We report here the complete genome sequence of a virulent *Staphylococcus epidermidis* siphophage, phage 6ec, isolated from the anterior nares of a human. This viral genome is 93,794 bp in length, with a 3′ overhang *cos* site of 10 nucleotides, and it codes for 142 putative open reading frames.

## GENOME ANNOUNCEMENT

The microbial ecology of the human anterior nares is complex, as it is a niche in which different bacterial genera, including staphylococci, compete for colonization (1). In some cases, this dynamic ecosystem leads to infectious diseases. Bacteriophages are expected to be an integral part of this anterior nare ecology. In order to better understand the factors modulating nasal colonization by staphylococci, we previously isolated several *Staphylococcus epidermidis* phages, belonging to the *Siphoviridae* and *Podoviridae* families, from the anterior nares of humans (2, 3). Here, we report the complete genome sequence and organization of one of the largest *Siphoviridae* bacteriophages isolated from the human anterior nare. The virulent phage 6ec has an icosahedral capsid (69 ± 1 nm in diameter) and unusually long noncontractile tail (362 ± 1 nm) (2).

The phage DNA was extracted using the Qiagen lambda maxi kit, as described previously (4), and its purity and quality were assessed on an agarose gel. Sequencing was done on an Illumina HiSeq 2000 at the University of Wisconsin-Madison Biotechnology Center’s sequencing facility, with 23,668-fold coverage and an average read length of 101 bp. The genome was assembled in GS *de novo* Assembler (version 2.9; 454 Life Sciences, Roche), and the assembly was confirmed by Consed (version 27.0) (5). To confirm the genome assembly, about 30 kb of the phage sequences spanning the entire genome were amplified and sequenced with independent site-specific PCRs and bidirectional Sanger sequencing, respectively. The physical ends were determined from the aligned read data using Consed. The putative open reading frames (ORFs) were predicted in DNA Master (see http://cobamide2.bio.pitt.edu/), putative functions were assigned using BLASTn and BLASTp (5), and a single tRNA was predicted using Aragorn (6). The potential regulatory elements were identified using PHIRE (7) and ARNold (http://rna.igmors.u-psud.fr/toolbox/arnold/).

The *S. epidermidis* phage 6ec genome is 93,794 bp, with a 10-bp 3′ overhang (AAGCGCCCCC) and a G+C content of 29.3%. It has 142 putative ORFs and a tRNA sequence coding for proline. Of the predicted ORFs, 26.8% had putative functions, and the rest were hypothetical proteins. The gene-coding potential of the genome was estimated to be 88.2%. The genes follow the general modular organization of other staphylococcal siphophage genomes (8), with genes coding for proteins involved in DNA metabolism, followed by genes involved in DNA packaging and morphogenesis. Interestingly, an integrase gene was also identified, although experimentally, the phage was lytic when propagated on *S. epidermidis* strain ATCC 35983 (2). This genome is twice the size of previously published staphylococcal phage genomes (93.8 kb versus 45 kb, respectively). Of note, an unpublished *S. epidermidis* phage (Sep9) genome of similar size (92.4 kb) is available in GenBank (GenBank accession no. NC_023582). The genome of 6ec has 72% query coverage (with 98% identity in the covered region) with the genome of phage Sep9. The genomes share synteny, although the opening of the phage Sep9 genome (in the current GenBank file) is distinct from that of phage 6ec.

### Nucleotide sequence accession number.

Phage 6ec has been deposited at the Félix d’Hérelle Reference Center for Bacterial Viruses (http://www.phage.ulaval.ca). The complete genome sequence of *S. epidermidis* phage 6ec has been deposited in GenBank under the accession no. KJ804259.
